# The Effect of 5-Aminolevulinic Acid on Cytochrome P450-Mediated Prodrug Activation

**DOI:** 10.1371/journal.pone.0131793

**Published:** 2015-07-16

**Authors:** Mai Miura, Kensuke Ito, Maiko Hayashi, Motowo Nakajima, Tohru Tanaka, Shun-ichiro Ogura

**Affiliations:** 1 Graduate School of Bioscience and Biotechnology, Tokyo Institute of Technology, 4259 B47 Nagatsuta-cho, Midori-ku, Yokohama, 226–8501, Japan; 2 SBI pharmaceuticals CO., LTD., Izumi Garden Tower 20F, 1-6-1, Roppongi, Minato-ku, Tokyo, 106–6020, Japan; Indian Institute of Toxicology Reserach, INDIA

## Abstract

Of late, numerous prodrugs are widely used for therapy. The hemeprotein cytochrome P450 (CYP) catalyzes the activation of prodrugs to form active metabolites. Therefore, the activation of CYP function might allow the use of lower doses of prodrugs and decrease toxicity. We hypothesized that the addition of 5-aminolevulinic acid (ALA), a precursor in the porphyrin biosynthetic pathway, enhances the synthesis of heme, leading to the up-regulation of CYP activity. To test this hypothesis, we treated a human gastric cancer cell line with ALA and determined the effect on CYP-dependent prodrug activation. For this purpose, we focused on the anticancer prodrug tegafur, which is converted to its active metabolite 5-fluorouracil (5-FU) mainly by CYP2A6. We show here that ALA increased CYP2A6-dependent tegafur activation, suggesting that ALA elevated CYP activity and potentiated the activation of the prodrug.

## Introduction

Cytochrome P450 (CYP) is a hemeprotein, virtually present in all organisms, that catalyzes the oxidation of endogenous and xenobiotic lipophilic substrates [1,2]. In general, the metabolism of drugs by CYPs mainly leads to drug inactivation and facilitates the elimination of drugs from the body [1,2]. However, CYPs also convert nontoxic prodrugs to cytotoxic metabolites [3,4], and many of these drugs are used to treat cancer. For example, the widely used anticancer prodrug cyclophosphamide is activated mainly by 4-hydroxylation reactions catalyzed by CYP2B6, CYP2C19, and CYP3A4 [4,5]. Further, CYP2A6 hydroxylates the anticancer prodrug tegafur, which converts it to the cytotoxic metabolite 5-fluorouracil (5-FU) [4,6,7].

The mono-oxygenase activities of CYPs require the heme prosthetic group [2]. Therefore, we assumed that hemeprotein activity might be affected by increasing or decreasing the intracellular levels of heme. 5-Aminolevulinic acid (ALA) is a well-known precursor in the protoporphyrin IX (PpIX) synthesis pathway leading to the synthesis of heme [8,9]. ALA is synthesized from glycine and succinyl CoA in the mitochondria and is then transported to the cytoplasm. Following some steps, ALA is returned to the mitochondria where it is converted to PpIX, which is converted to heme by binding ferrous ion. ALA synthesis is the rate-limiting step of this pathway [10]. Therefore, the addition of ALA to cells might promote the biosynthesis of heme, and we hypothesized that activating heme synthesis increases the expression and activity of hemeproteins.

We previously reported that the expression and activity of the hemeprotein cytochrome c oxidase is elevated in ALA-treated cells [11,12]. Moreover, we found that the expression of PEPT1, a transporter involved in the uptake of ALA, is higher in tumor tissues compared with that in normal tissues [13]. Therefore, we concluded that it is likely that hemeproteins are specifically activated in tumor cells in the presence of ALA. Because the activities of hemeprotein enzymes, such as CYP, require the heme prosthetic group [2], we hypothesized that ALA enhances heme biosynthesis, leading to increased CYP activity. Therefore, we examined the effect of ALA on CYP-dependent anticancer prodrug activation in a human gastric cancer cell line. For this purpose, we focused on the anticancer prodrug tegafur, which is converted to cytotoxic 5-FU by CYP2A6. Further, we utilized sodium ferrous citrate (SFC) as the source of ferrous ion, which is required to convert PpIX to heme.

Our results indicate that the addition of ALA and SFC to cultured cells elevated CYP activity and potentiated prodrug activation, suggesting the potential of ALA for use in CYP-dependent prodrug therapy. This approach might facilitate using lower doses of prodrugs and decrease toxic side effects.

## Materials and Methods

### Biochemicals

ALA hydrochloride and SFC were purchased from Cosmo Oil Co., Ltd. (Tokyo, Japan). RPMI-1640 medium, antibiotic-antimycotic (ABAM, penicillin-streptomycin-amphotericin B mixture), and NADPH were obtained from Nacalai Tesque (Kyoto, Japan). Fetal bovine serum (FBS) was purchased from Equitech-Bio, Inc. (Kerrville, Texas). Tegafur was purchased from LKT Laboratories, Inc. (St. Paul, MN). All other chemicals were analytical grade.

### Cell culture

The human gastric cancer cell line MKN28 was provided by Dr. Endo (Kanazawa University, Ishikawa, Japan). MKN28 was established from metastatic foci to lymph nodes [14]. The cells were grown in RPMI-1640 medium supplemented with 10% FBS and ABAM at 37°C in an incubator with a controlled humidified atmosphere containing 5% CO_2_.

### Detection of mRNA levels using RT-PCR

Total RNA was isolated using a NucleoSpin RNA II kit (Macherey-Nagel, Düren, Mannheim, Germany) and transcribed to cDNA using a PrimeScript RT kit (TaKaRa Bio, Otsu, Japan). First-strand cDNAs encoding CYP2A6 and GAPDH were amplified using PCR in a Thermal Cycler Dice Mini (TaKaRa Bio, Otsu, Japan) with the primer sets as follows: CYP2A6 [15], forward 5′-TCAAAGGCTATGGCGTGGTA-3′ and reverse 5′- CTGACGGTCTCGGTGCCCC-3′; GAPDH, forward 5′-GCACCGTCAAGGCTGAGAAC-3′ and reverse 5′-TGGTGAAGACGCCAGTGGA-3′. The PCR reactions comprised incubation at 95°C for 5 min (“hot start”) followed by 35 cycles each at 95°C for 30 s and 60°C for 1 min. The resulting amplicons were separated electrophoretically through 2.5% agarose gels, stained with ethidium bromide, and visualized using ultraviolet light.

### Analysis of heme levels

Intracellular heme levels were determined using a published fluorometric method [16,17] in which the heme contained within a hemeprotein is converted to its fluorescent porphyrin derivative by incubation with oxalic acid. Cells were seeded in 6-well plates, allowed to attach overnight in the culture medium, and then incubated for 8 h in the culture medium containing 1 mM ALA alone or together with 0.5 mM SFC. The cells were rinsed twice with 1 ml of PBS and lysed in 600 μl of 0.02 M NaOH. This lysate (60 μl) was mixed with 940 μl of 2.0 M oxalic acid and heated to 95°C for 30 min to dissociate iron from heme to generate PpIX. Samples were then centrifuged for 10 min at 1,000 × *g* at 4°C to remove debris. The fluorescence of PpIX in the supernatant was measured using excitation and emission wavelengths of 405 nm and 637 nm, respectively, and the data were normalized to the protein concentration of each sample.

### MTT assay

The combined effect of ALA and tegafur on MKN28 cells was determined using an MTT assay. Cells were seeded in 96-well plates (0.3 × 10^4^ cells per well) and cultured at 37°C for 24 h in an atmosphere containing 5% CO_2_. Increasing concentrations of tegafur were added to the culture medium containing 0.3 mM NADPH with or without 1.0 mM ALA and 0.5 mM SFC, and the cells were further incubated for 96 h. Viable cells were quantified using an MTT assay as previously described [18].

### Analysis of 5-FU synthesis

Cells were seeded in 6-well plates, allowed to attach overnight in the culture medium, and then incubated for 8 h in a culture medium containing 1 mM tegafur, 0.3 mM NADPH, 1 mM ALA, and 0.5 mM SFC. The culture medium was collected for HPLC analysis. Cells were rinsed with 1 ml of PBS, treated with 400 μ1 of 0.1 M NaOH, and the lysates and culture medium were extracted using perchloric acid: methanol (1:1, v/v) as descrived previously [19]. The mixture was centrifuged at 12,000 rpm for 10 min to remove protein, and the supernatant was subjected to HPLC analysis as previously described [6]. 5-FU formation was determined using HPLC with a C18 (5 μm) analytical column (250 × 4.6-mm i.d., Mightysil RP-18 GP, Kanto Chemical, Tokyo, Japan) at 35°C. Compounds were eluted at a flow rate of 1.2 ml/min with a solution containing 3% CH_3_OH and 20 mM NaClO_4_ (pH 2.5). The eluent was monitored at 270 nm using SPD-20V UV-Vis detectors (Shimadzu, Kyoto, Japan). Tegafur and 5-FU levels were normalized to the intracellular protein level determined using the Bradford assay (Quick Start Bradford protein assay, Bio-Rad Laboratories, Inc., CA).

### Inhibitor experiments

Cells were incubated in culture medium containing inhibitors (5 μM Ko143, 10 μM fumitremorgin C (FTC), or 100 μM cholic acid), 1 mM tegafur, 0.3 mM NADPH, 1 mM ALA, and 0.5 mM SFC and were subsequently analyzed for heme content and viability using the assays described above.

## Results

### The effect of ALA on the expression of CYP2A6 mRNA and heme

**Fig 1:**
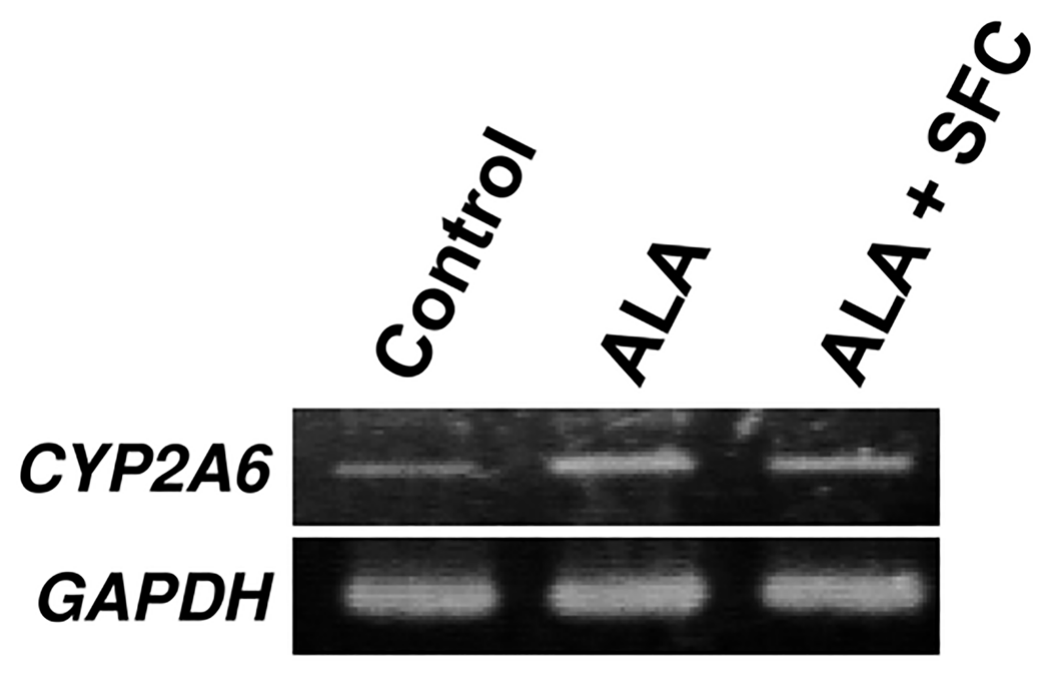
Expression level of *CYP2A6* mRNA. *CYP2A6* mRNA levels were determined by RT-PCR after incubating MKN28 cells with 1.0 mM ALA, 0.5 mM SFC, or both for 8 h. GAPDH is used as internal control.

To evaluate the effect of ALA and SFC on the human gastric cancer cell line MKN28, we determined the levels of the mRNA encoding CYP2A6, which converts tegafur to 5-FU. After ALA treatment for 8 h, *CYP2A6* mRNA levels increased compared with those of the control (Fig 1). Similarly, *CYP2A6* mRNA levels increased when ALA and SFC were added together (Fig 1).

**Fig 2:**
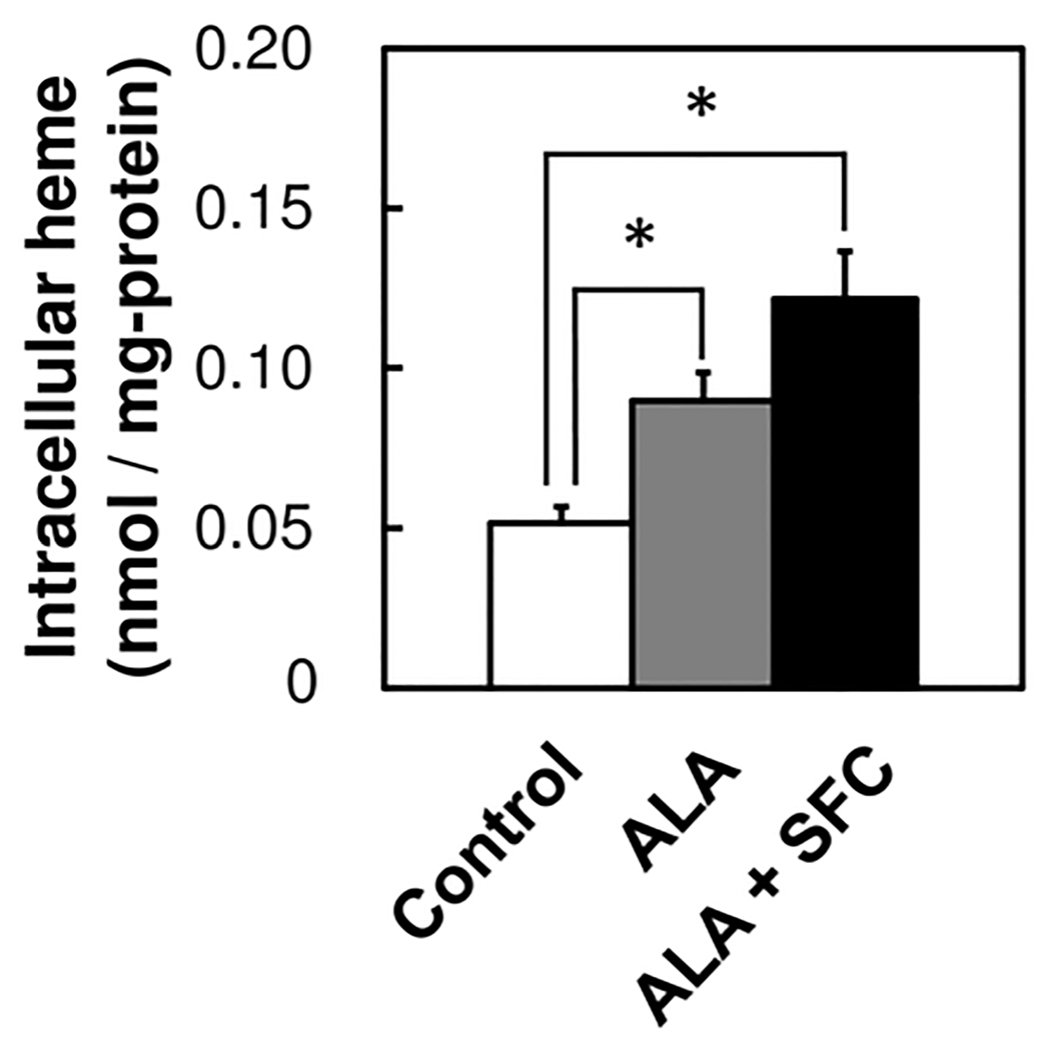
The effect of ALA / SFC on the intracellular heme level. The intracellular heme level was determined by fluoremetric method after incubating MKN28 cells with 1.0 mM ALA, 0.5 mM SFC, or both for 8 h. Data are expressed as means ± S.D. in three independent experiments. Statistical significance of difference is indicated by *p<0.01 determined with Student’s t-test.

Intracellular heme levels were determined after incubating MKN28 cells with ALA alone or together with SFC for 8 h (Fig 2). The intracellular level of heme increased in the presence of ALA and increased further in the presence of both ALA and SFC (Fig 2). We conclude that the data can be explained as follows: ALA administration increased PpIX synthesis and contributed to increased intracellular heme levels, and SFC, as a source of iron ion, enhanced heme synthesis further in the presence of ALA. These data indicate that the addition of ALA or both ALA and SFC increased the synthesis and activity of CYP, which facilitated increased heme synthesis.

### The effects of ALA and tegafur

**Fig 3:**
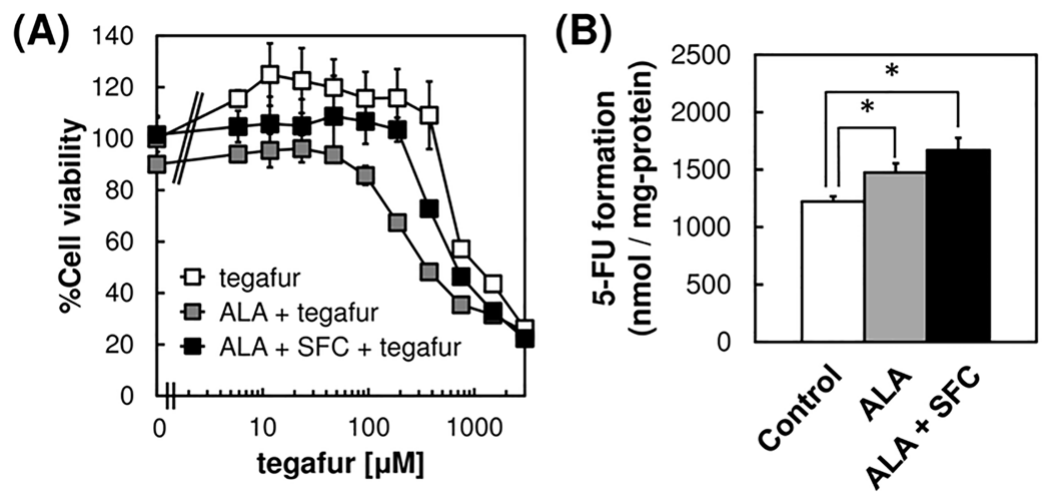
Activation of tegafur to 5-FU in the presence of ALA / SFC. (A) Growth inhibition of tegafur was determined using an MTT assay after incubating MKN28 cells with 1.0 mM ALA alone or together with 0.5 mM SFC for 96 h. Data are percent growth rate relative to the drug-free controls. (B) 5-FU formation was determined using HPLC. Cells were incubated for 8 h in a culture medium containing 1.0 mM tegafur, 0.3 mM NADPH, 1.0 mM ALA, and 0.5 mM SFC. Data are expressed as means ± S.D. in four (A) or three (B) independent experiments. Statistical significance of difference is indicated by *p<0.01 determined with Student’s t-test.

Because our results suggest that ALA and SFC increased CYP2A6 expression and heme synthesis, we determined whether tegafur was cytotoxic to MKN28 cells in the presense of ALA and SFC. Tegafur treatment combined with ALA alone or together with SFC increased cytotoxicity compared with tegafur treatment alone (Fig 3a). These results suggest that tegafur treatment combined with ALA or both ALA and SFC elevated CYP2A6 activity and potentiated prodrug activation. Consistent with this conclusion was the finding that the levels of 5-FU were increased in the presence of ALA and tegafur (Fig 3b). The increase in CYP2A6 activity may be attributed to the induction of the synthesis of *CYP2A6* mRNA and intracellular heme by ALA. Moreover, the levels of 5-FU increased when cells were treated with both ALA and SFC (Fig 3b). We attribute this potentiation to the increase of intracellular heme levels by the treatment of the cells with SFC.

### The effect of inhibitors of transporter activity on tegafur activation

**Fig 4:**
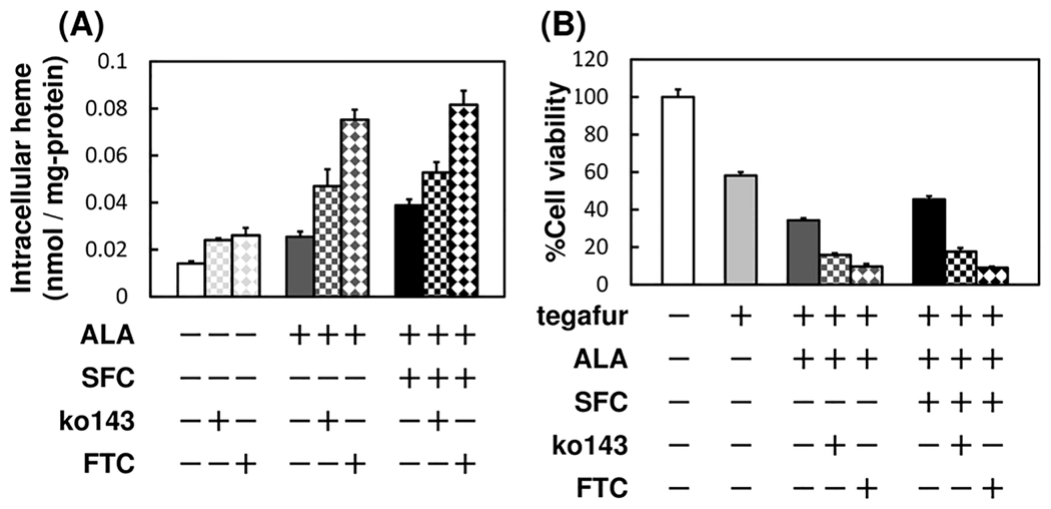
The effect of inhibitors of ABCG2 transporter activity on tegafur activation. (A) The effect of ABCG2 inhibitor on heme synthesis in the presence of ALA alone or together with SFC. Cells were incubated in culture medium containing inhibitors (5 μM Ko143, 10 μM FTC, or 100 μM cholic acid), 1.0 mM ALA, and 0.5 mM SFC for 8 h and were subsequently analyzed for heme content. (B) The effect of ABCG2 inhibitor on the cytotoxic effect of tegafur in the presence of ALA alone or together with SFC. Cells were incubated in culture medium containing inhibitors (5 μM Ko143 or 10 μM FTC), 1.0 mM tegafur, 0.3 mM NADPH, 1.0 mM ALA, and 0.5 mM SFC for 96 h and were subsequently analyzed for viability. Data are expressed as means ± S.D. in three (A) or four (B) independent experiments.

Our results indicate that treatment of MKN28 cells with a combination of ALA and SFC contributed to tegafur activation by its CYP2A6-mediated conversion to 5-FU. Accordingly, efflux of PpIX or 5-FU would be expected to attenuate the potentiating effect of ALA and SFC. To address this possibility, we inhibited the export of PpIX or 5-FU in an attempt to enhance the effects of ALA and SFC. PpIX is exported from the cell by ABCG2 [13, 20–22]. Therefore, we treated MKN28 cells with the ABCG2 inhibitors Ko143 and FTC and determined the amount of intracellular heme and cytotoxicity of tegafur in the presence of ALA and SFC. There was no significant change in the intracellular heme level in the absence of ALA and SFC with or without the ABCG2 inhibitors. In contrast, treating cells with ALA alone or combined with SFC in the presence of ABCG2 inhibitors markedly increased intracellular heme levels (Fig 4a). Moreover, the cytotoxic effect of tegafur in the presence of Ko143 or FTC was strongly enhanced by ALA alone or together with SFC (Fig 4b). Taken together, these results suggest that inhibiting ABCG2-mediated drug efflux increased the accumulation of PpIX in the cytoplasm and up-regulated subsequent heme synthesis and CYP2A6 activity.

**Fig 5:**
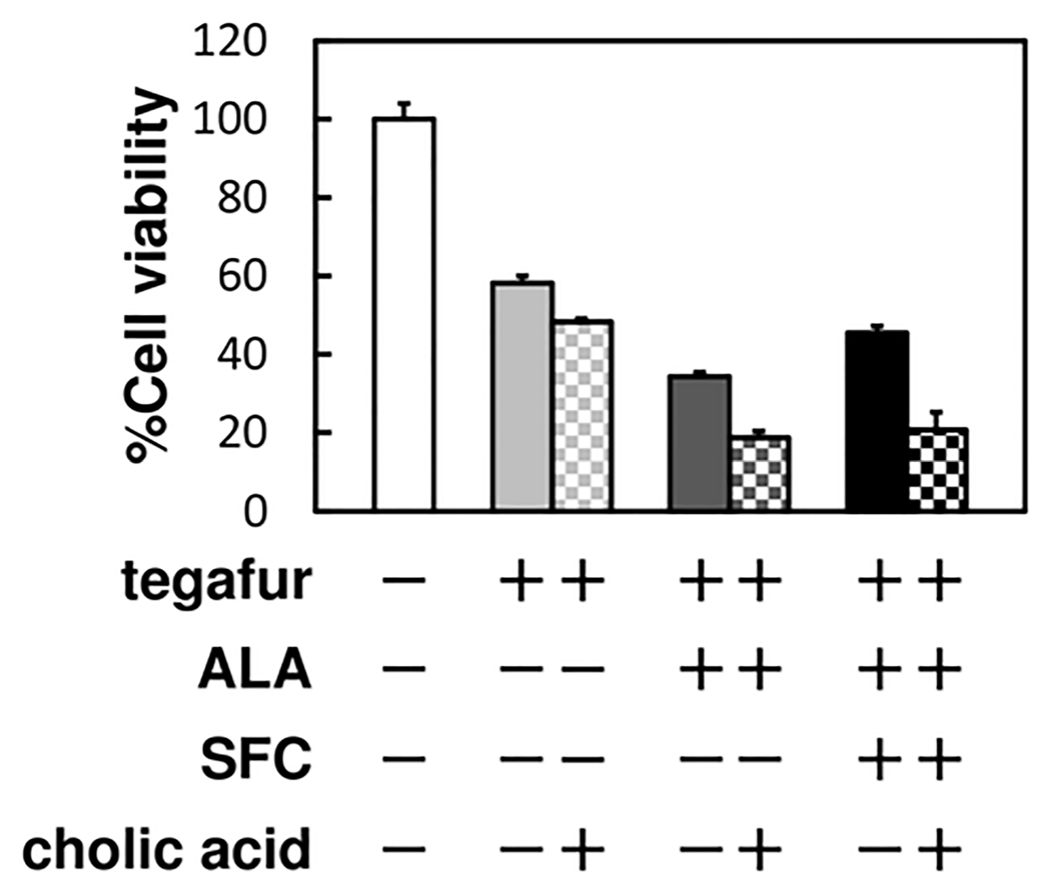
The effect of cholic acid on tegafur activation. Cells were incubated in culture medium containing 100 μM cholic acid, 1.0 mM tegafur, 0.3 mM NADPH, 1.0 mM ALA and 0.5 mM SFC for 96 h and were subsequently analyzed for viability. Data are expressed as means ± S.D. in four independent experiments.

In contrast, 5-FU or its analogs may be exported by specific ABC transporters [23–25]. We found that colic acid, which inhibits certain ABC transporters [26–28], potentiated the cytotoxic effect of tegafur in the presence of ALA alone or with SFC (Fig 5).

## Discussion

Effective activation of CYP is useful for converting prodrugs to their active therapeutic metabolites. In the present study, we used the human gastric cancer cell line MKN28 to show that ALA and SFC activated CYP and increased the cytotoxicity of the anticancer prodrug tegafur. These findings indicate that tegafur was activated in this cancer cell line.

This conclusion is supported by several observations. First, *CYP2A6* expression was induced in MKN28 cells treated with ALA and SFC and was likely mediated by a heme-dependent transcription factor. Second, the addition of ALA and SFC increased the intracellular heme levels. Enhanced *CYP2A6* expression and heme biosynthesis indicate that ALA and SFC treatment might contribute to the elevation of CYP activity by increasing the levels of apo-CYP and its heme prosthetic group. Third, we show that treating MKN28 cells with tegafur together with ALA and SFC increased the formation of 5-FU from tegafur and enhanced the cytotoxicity. Because we selected the concentration range of ALA and SFC that does not affect cell viability, enhanced cytotoxicity of tegafur in the presence of ALA and SFC may be attributed to the up-regulation of CYP activity. Taken together, these data support the use of a cancer treatment strategy that employs ALA and SFC to activate CYP-dependent prodrugs. This is the first report, to our knowledge, showing that a CYP-dependent prodrug is activated in a cell line treated with ALA and SFC.

Moreover, the addition of transporter inhibitors potentiated the effect of ALA and SFC on intracellular heme levels and tegafur cytotoxicity. Specifically, when we used FTC and Ko143 to inhibit the ABCG2-mediated efflux of PpIX, tegafur cytotoxicity was markedly increased. Moreover, when we treated cells with cholic acid to efflux 5-FU, tegafur was highly cytotoxic. These results suggest that the utilization of ALA, SFC, and a transporter inhibitor might activate prodrugs more effectively, which would enhance cancer therapy by allowing the use of lower doses of prodrugs with the added benefit of reducing toxicity.

There are many approaches for elevating CYP activities to effectively activate prodrugs. For example, Waxman et al. reported the use of oxazaphosphorines as CYP inducers that effectively activate anticancer prodrugs [29]. Waxman et al. reported subsequently a gene therapy strategy using canine cytochrome P450 2B11 to activate anticancer prodrugs [30]. These approaches are quite similar to ours, although those of Waxman et al. seem to have low tumor selectivity. Moreover, ALA-dependent porphyrin accumulation is tumor specific [13], and ALA causes no side effects on normal cells [9]. Therefore, our strategy offers significant advantages for tumor therapy using prodrugs. Further studies are required to assess the applicability of our strategy *in vivo* using experimental xenograft models and clinical tumor specimens.
